# Effects of Septum Resection for Secondary Infertility on Subsequent Reproductive Outcomes of *in vitro* Fertilization–Intracytoplasmic Sperm Injection

**DOI:** 10.3389/fmed.2022.765827

**Published:** 2022-02-03

**Authors:** Huixiao Chen, Ping Sun, Na Zhang, Shangge Lv, Yongzhi Cao, Lei Yan

**Affiliations:** ^1^School of Medicine, Cheeloo College of Medicine, Shandong University, Jinan, China; ^2^Center for Reproductive Medicine, Cheeloo College of Medicine, Shandong University, Jinan, China; ^3^Department of Gynecology and Obstetrics, Liaocheng People's Hospital, Liaocheng, China; ^4^Shandong Provincial Hospital Affiliated to Shandong First Medical University, Jinan, China

**Keywords:** secondary infertility, uterine septum, reproductive outcomes, *in vitro* fertilization, intracytoplasmic sperm injection IVF-ET, *in-vitro* fertilization-embryo transfer ICSI, intracytoplasmic sperm injection

## Abstract

**Objective:**

To assess the effect of uterine septum resection on reproductive outcomes of *in vitro* fertilization (IVF) / intracytoplasmic sperm injection (ICSI) in patients with secondary infertility complicated with uterine septum.

**Methods:**

A retrospective cohort study included 269 patients. Surgical group included 169 patients with secondary infertility complicated with uterine septum, who underwent 252 embryo-transfer (ET) cycles following septum resection. Control group consisted of 100 patients with secondary infertility and uterine septum, who underwent 178 ET cycles. Cumulative pregnancy rate and cumulative live birth rate after one complete assisted reproductive technology (ART) cycle were the primary outcomes.

**Results:**

The results showed that the cumulative pregnancy rate was higher in the surgery group, and statistically significant difference was observed in the cumulative pregnancy rate between the two groups (71.0 vs. 59%, *P* = 0.044). In fresh ET cycle, no statistically significant difference between the two groups was evident (54.9 vs. 40.6%, *P* = 0.061). Statistical analysis of other results of the fresh ET cycle did not differ significantly between the two groups. In terms of frozen embryo transfer (FET) cycle outcomes, the clinical pregnancy rate and delivery rate in surgery group were 52.7 and 38.2%, respectively, which were significantly higher than those in the control group (38.2 and 22.5%, respectively) (*P* = 0.028 and *P* = 0.011).

**Conclusion:**

The reproductive outcomes of IVF/ICSI after septum resection in patients with secondary infertility were better than that in the untreated group, suggesting that uterine septum resection can be performed in patients with uterine septum combined with infertility to improve their reproductive outcomes.

## Introduction

Uterine malformations are the result of abnormal formation, differentiation, and fusion of müllerian or accessory renal ducts during the fetal period, and are believed to be one of the many causes of female infertility (1). Among all the congenital uterine deformities, uterine septum is the most common congenital anomaly of the reproductive tract, and it is described as a uterus with a normal contour and an internal septum at the fundal midline. Between 11 and 14 weeks of gestation, the thickness of the uterine septum exceeded 50% of the uterine wall thickness because of malabsorption of bilateral müllerian ducts (2–4). Previously, the gold standard was a combination of laparoscopy and hysteroscopy. In recent decades, imaging technology, for example, hysterosalpingography (HSG), magnetic resonance imaging (MRI), sonohysterography, saline infusion sonography (SIS), and ultrasonography (US), preferably transvaginally (TVS), has advanced rapidly with less invasiveness (5). However, studies on how to best diagnose a septum are limited by sample sizes. There are no clear guidelines for the optimal diagnosis of the uterine septum. A guideline suggests that imaging with hysteroscopy should be used to diagnose uterine septa, which is better than laparoscopy combined with hysteroscopy, because it is less invasive (Grade B). Imaging and an experienced clinician are required to determine the septum diagnosis (6). In clinical practice, uterine septum is usually classified into incomplete and complete uterine septum. There are many proposed classification systems for müllerian anomalies, and the American Fertility Society (AFS) Classification from 1988 has been the most recognized and utilized. In the AFS classification, uterine septum is represented as simple drawings (class Va: Complete uterine septum and class Vb: Partial uterine septum), without providing strict parameters to define septate configurations (7). In 2021, Samanth et al. published a new paper on müllerian anomalies, partially updating the uterine septum classification, and specific numerical values were given. They defined septate uterus as having an endometrial septum length of >1 cm, as measured from the bicornual line, with the leading edge of the septum having an angle of <90° (8).

A multitude of studies have so far reported the association between uterine septum and the high rate of miscarriage, recurrent pregnancy loss, and premature delivery (9–11). However, the exact mechanism by which the uterine septum causes subfertility is not completely understood. The insufficiency of the cervix and the diminished size of the uterine cavity are considered to be the two leading causes of subfertility (12, 13). In addition, many literatures have reported different research results on the composition of the uterine septum, which may exert negative effects on fertility and lead to adverse reproductive outcomes (14). For women with uterine septum, the reproductive history of recurrent abortions or fatal loss are considered as the absolute and main indication for the treatment (15). Limited researches have shown that the surgical correction of uterine abnormalities can improve live birth rate and pregnancy rate in infertile women (9, 16, 17). In a retrospective multicentral study, the researchers compared the *in vitro* fertilization (IVF) results in women with untreated uterine septum with women in general, and found significantly lower rate of implantation and pregnancy in the untreated group.

Several researches based on self-control design have examined the reproductive outcomes before and after treatment of the uterine septum and found that septate uterus, subseptate uterus, and arcuate uterus increased the risk of spontaneous abortion, and negatively affected implantation, thereby reducing the live birth rate during IVF/intracytoplasmic sperm injection (ICSI) (9, 18, 19). According to a number of literatures reported, hysteroscopic septum resection is associated with an improvement in live birth rate in women with infertility or prior pregnancy loss. However, there is no prospective randomized controlled trial (RCT) to prove the effectiveness of the surgery. Because it is unethical to assign patients to the “no treatment” group knowing that malformation is one of the causes of abortion (20).

Very few studies have known to be conducted on whether uterine septum resection can be performed in patients with secondary infertility, which is defined as a previous history of pregnancy followed by 12 consecutive months of non-pregnancy without contraception. In addition, there is no clear evidence on the possible effectiveness of hysteroscopic metroplasty in women with secondary infertility. In order to determine whether hysteroscopic metroplasty in such patients improves the reproductive outcomes, we conducted a retrospective cohort study to evaluate the effect of septoplasty as therapy for secondary infertility in women with uterine septum.

## Materials and Methods

The retrospective cohort study was conducted at the Reproductive Hospital affiliated to Shandong University. We collected the medical records of patients with secondary infertility as well as uterine septum, who were admitted to our hospital for IVF/ICSI treatment between 2009 and 2019, and performed further screening according to the inclusion and exclusion criteria.

### Participants

All patients included in this study suffered from secondary infertility and uterine septum. Among these patients, 96 patients experienced live birth, while others had an induced or medical abortion as a result of an unplanned pregnancy or had an ectopic pregnancy. We excluded patients with recurrent miscarriage. Patients included in this study underwent a complete set of pre-assisted reproductive technology (ART) examination including base estrogen, progestin, serum follicle-stimulating hormone (FSH), luteinizing hormone (LH) levels, serum prolactin, thyroid-stimulating hormone levels, and gynecological examination. Hysteroscopy and transvaginal ultrasound are also necessary which could help doctors understand the condition of the uterine cavity. Nearly all uterine septum in this study were diagnosed by means of hysteroscope and transvaginal ultrasonic. Diagnostic hysteroscopy, which has the highest sensitivity, specificity, and positive and negative predictive values, was part of the infertility workup of patients, and hysteroscopy is considered the gold standard for assessing intrauterine abnormalities (6, 21). When patients underwent hysteroscopy, the uterine septum was divided into incomplete septum and complete septum by an experienced physician according to the up-to-date classification criteria at the time. Postoperative B ultrasound or hysteroscopy was performed to examine the shape and recovery of the uterine cavity. Inclusion criteria were as follows: (1) age 18–45 years; (2) patients suffering from secondary infertility and uterine septum; (3) a full cycle of IVF/ICSI has been performed. Exclusion criteria were: (1) a history of recurrent abortion; (2) severe systemic disease; (3) presence of a chromosomal abnormality in the male or female partner; (4) congenital uterine malformation other than the septate uterus, e.g., unicornuate uterus and bicornuate uterus; (5) presence of uterine or pelvic disease, such as severe intrauterine adhesions, uterine adenomyosis, and untreated hydrosalpinx; (6) participation in a donor oocyte program or presence of a preimplantation genetic test (PGT); and (7) giving up the embryo transfer and treatment midway or no follow-up information availability.

### Surgical Technique

Patients in the surgery group received hysteroscopic division of the uterine septum before IVF cycles. The surgery was performed under general anesthesia during the follicular phase of the menstrual cycle. We aimed to create triangular and symmetric uterine cavity as much as possible, and patients were re-examined 1–2 months after surgery to evaluate the postoperative uterine cavity. Examination reports from other first-class hospitals were admitted. If a residual septum of >1.5 cm was observed, hysteroscopic surgery was repeated.

### Outcome Measures

The primary outcomes of our study are the cumulative pregnancy rate and the cumulative live birth rate after one complete assisted reproductive technology (ART), in other words, the best reproductive outcome of all the cycles, fresh and frozen, that the patient underwent. Cumulative pregnancy rate was calculated by the number of first clinical pregnancies from a single IVF/ICSI cycle, including all fresh or frozen-thawed embryo-transfer (ET) cycles generated from the index cycle, divided by the total number of women who received treatment. Here, clinical pregnancy is defined as a pregnancy diagnosed by ultrasonographic visualization of one or more gestational sacs or definitive clinical signs of pregnancy. In addition to intra-uterine pregnancy, clinical pregnancy includes a clinically documented ectopic pregnancy (22). Cumulative live birth rate was calculated by the number of first live births generated from a single IVF/ICSI cycle divided by the number of women who received treatment. Live birth is defined as the complete expulsion or extraction from a women of a product of fertilization, irrespective of the duration of the pregnancy, which, after such separation, breathes or any other evidence of life should be showed (22). Secondary outcome measures were: (1) clinical pregnancy rate; (2) clinical live birth rate; (3) biochemical pregnancy rate; (4) premature live birth rate; (5) term delivery rate; (6) post-term delivery rate; (7) ectopic pregnancy rate; and (8) miscarriage rate. Biochemical pregnancy is defined as a pregnancy diagnosed only by the detection of human chorionic gonadotropin β-subunit (β-hcg) in serum or urine, and biochemical pregnancy rate was calculated as the number of women, whose β-hcg level was more than 10 mIU/mL at 14 days after transfer, divided by the number of embryo transfer (ET) cycles. Preterm live birth is a birth that takes place after 22 weeks and before 37 completed weeks of gestational age, and preterm delivery rate is the number of deliveries occurring between 28 and 37 weeks divided by the number of total deliveries. Post-term birth is a live birth or stillbirth that takes place after 42 completed weeks of gestational age and post-term birth rate is the number of deliveries that occur after 42 completed weeks of gestational age weeks divided by the number of total deliveries. The definition of ectopic pregnancy is a pregnancy outside the uterine cavity, diagnosed by ultrasound, surgical visualization, or histopathology (22). Ectopic pregnancy rate was calculated by dividing the number of cycles of a pregnancy with the embryo plant outside the uterine cavity by the number of clinical pregnancy cycles. The secondary outcomes were compared both in fresh embryo transfer (ET) and frozen embryo transfer (FET) cycles between the surgical group and the expectant group. According to the American Fertility Society classification (AFS) of Müllerian duct anomalies, we further classified the uterine septum (class Va: Complete uterine septum and class Vb: Partial uterine septum) (7) and compared the reproductive outcomes between the different types of the septum.

### Statistical Analysis

SPSS 26.0 software (IBM Corp., Armonk, NY, USA) was used for statistical analysis of relevant data. We applied the *t*-test to obtain group comparisons of measurement data. If measurement data were normally distributed, they were expressed as mean ± standard deviation, and the measurement data that did not conform to a normal distribution were represented by median (25th percentile, 75th percentile). The Mann–Whitney *U*-test was used for comparisons between the two groups. Counting data were expressed as percentage, and intergroup comparisons were performed using the chi-square test or Fisher's exact test.

### Ethics Statement

This study was approved by the Institutional Review Board (IRB) of the Reproductive Hospital affiliated to Shandong University (2019-119). Written informed consent was obtained from the participants when they presented for IVF-ICSI treatment.

## Results

After careful screening, 269 patients were eventually enrolled in this study. Among them, about 100 patients preferred to undergo transplantation of embryo rather than surgical treatment of the uterine septum, and we defined them as control (expectant) group, who underwent a total of 178 cycles of ET. The remaining 169 patients with a confirmed uterine septum decided to undergo surgery before ET, and they were defined as the surgical group. The patients in this group underwent a total of 252 cycles of ET. The total number of ET cycles per patient included both fresh and frozen attempts.

We compared the demographic characteristics of the control group and the surgical group, and the results are presented in Table 1. As illustrated in the table, no significant difference was observed in age, BMI, duration of infertility, basal FSH, basal LH, basal estradiol (E2), basal prolactin (P), basal testosterone (T), total antral follicle counting (AFC), endometriosis, paternal age, semen density, semen motility rate, normal semen morphology rate, and the type of uterine septum. However, the rate of patients who had the experience of live birth in the control group was much higher than in the surgical group, and statistical differences were observed. The characteristics of controlled ovarian stimulation and embryological results between the control group and the surgical group were also compared, and the results are presented in Table 2. There were no significant differences between the two groups with respect to ovarian stimulation regimens and the proportions of ICSI treatment. The two groups did not differ significantly in the total gonadotropin (Gn) days, total Gn dosage, endometrial thickness on trigger-day, and >14 mm oocytes on trigger-day. The obtained oocytes, two pronuclei, high-quality embryos, and the number of embryos transferred were also comparable.

**Table 1:** Demographic data.

	**Control group** **(***N*** = 100)**	**Surgical group** **(***N*** = 169)**	***P*** **-value**
Age (years)	34 (30–37)	33 (29–36)	*P* = 0.055
BMI (kg/m^2^)	23.09 (21.22–25.38)	23.44 (21.19–26.84)	*P* = 0.386
Infertility duration (years)	2.50 (1–4.75)	3.0 (2.00–5.00)	*P* = 0.174
Total AFC, *n*	12 (8.5–18)	14 (10–18)	*P* = 0.088
Endometriosis	1 (1%)	2 (1.2%)	*P* = 1.000
Basal FSH (mIU/mL), Mean ± SD	6.83 ± 1.71	6.82 ± 2.43	*P* = 0.963
Basal LH (IU/L)	4.81 (3.82–6.66)	4.61 (3.52–6.26)	*P* = 0.683
Basal E2 (pg/mL)	38.2 (29.6–48.2)	36.2 (29.7–49.6)	*P* = 0.473
Basal PRL (ng/mL)	14.37 (11.15–20.66)	13.89 (10.96–19.46)	*P* = 0.347
Basal T (ng/dL)	34.21 (16.46–32.18)	23.48 (17.57–33.27)	*P* = 0.867
Uterine septum type			*P* = 0.410
Partial uterine septum, *n* (%)	88 (88%)	154 (91.1%)	
Complete uterine septum, *n* (%)	12 (12%)	15 (8.9%)	
Live birth experience	52 (52%)	43 (25.4%)	*P* = 0.000*
Paternal age (years)	35 (31–38)	33 (30–37)	*P* = 0.077
Semen density (*10^6^/ml)	49 (28.3–80.5)	40 (23.9–69.0)	*P* = 0.122
Sperm motility rate (%)	53.8 ± 22.7	53.2 ± 20.4	*P* = 0.844
Normal semen morphology rate (%)	4.44 (2.59–7.69)	4.29 (2.00–6.87)	*P* = 0.173

*SD, standard deviation; BMI, body mass index; FSH, follicle-stimulating hormone; LH, luteinizing hormone; E2, estradiol; PRL, prolactin; T, testosterone; AFC, antral follicle counting*.

* *P < 0.05*.

**Table 2:** Characteristics of IVF-ICSI stimulation cycles and embryological results.

	**Control group** **(***N*** = 100)**	**Surgical group** **(***N*** = 169)**	***P*** **-value**
Number of IVF/ICSI cycles, *n*	100	169	
ICSI, *n* (%)	7 (7%)	18 (10.7%)	*P* = 0.319
Ovarian stimulation protocol			*P* = 0.122
GnRH agonist long protocol, *n* (%)	51 (51%)	107 (63.3%)	
GnRH agonist short protocol, *n* (%)	32 (32%)	35 (20.7%)	
GnRH antagonist protocol, *n* (%)	11 (11%)	21 (12.4%)	
Others, *n* (%)	6 (6%)	6 (3.6%)	
Total Gn time (Day)	11 (10–12)	11 (10–13)	*P* = 0.539
Total Gn dosage (IU)	1,800 (1,368.75–2,400)	1,875 (1,387.50–2,400)	*P* = 0.689
EM thickness on trigger-day (mm)	1 (0.83–1.10)	1 (0.80–1.10)	*P* = 0.975
>14 mm Oocytes on trigger-day	9 (6.5–13.5)	9 (7–13)	*P* = 0.388
Oocytes obtained	9 (6–15.5)	9 (7–13)	*P* = 0.521
2PN embryos	6.5 (4–9)	10 (7–15)	*P* = 0.918
High-quality embryos	4 (2–6)	4 (2–6)	*P* = 0.628
Number of embryos transferred	1 (1–2)	1 (1–2)	*P* = 0.259

*IVF, in vitro fertilization; ICSI, intracytoplasmic sperm injection; Gn, gonadotropin; GnRH, gonadotropin-releasing hormone; EM, endometrium; PN, pronucleus*.

We retrospectively compared the reproductive outcomes of fresh and frozen ET cycles between the surgical group and control group, and the results are presented in Tables 3, 4, respectively. According to the final results of fresh ET cycles, a total of 76 fresh ET cycles were performed in the control group and 121 fresh ET cycles were performed in the surgery group. The clinical pregnancy rate of the surgical group tended to be higher than that of the control group, but no significant statistical difference was observed between the two groups (54.9 vs. 40.6%, *P* = 0.061). Statistical analysis for other outcomes of fresh ET cycles showed that clinical live birth rate, miscarriage rate, ectopic pregnancy rate, chemical pregnancy rate, preterm live birth rate, term delivery rate, and post-term delivery rate were comparable between the two groups. The reproductive outcomes of FET are presented in Table 4. There were 102 FET cycles in the control group and 131 FET cycles in the surgical group. The clinical pregnancy rate and delivery rate in the surgical group were 52.7 and 38.2%, respectively, which were significantly higher than those in the control group (38.2 and 22.5%, respectively, *P* = 0.028 and *P* = 0.011). Other results were not significantly different between the two groups (Table 4).

**Table 3:** Reproductive outcomes calculated per fresh ET cycle.

	**Control group** **(***N*** = 100)**	**Surgical group** **(***N*** = 169)**	***P*** **-value**
Number of ET cycles, *n*	76	121	
Clinical pregnancy rate, % (*n*)	40.6% (28/76)	54.9% (62/121)	*P* = 0.061
Abortion rate, % (*n*)	10.7% (3/28)	25.8% (16/62)	*P* = 0.104
Ectopic pregnancy rate, % (*n*)	0 (0/28)	1.6% (1/62)	*P* = 1.000
Live birth rate, % (*n*)	32.9% (25/76)	37.2% (45/121)	*P* = 0.540
Premature delivery rate, % (*n*)	6.6% (5/76)	3.3% (4/121)	*P* = 0.311
Term delivery rate, % (*n*)	26.3% (20/76)	33.1% (40/121)	*P* = 0.317
Post-term birth rate, % (*n*)	0 (0/76)	0.8% (1/121)	*P* = 1.000
Biochemical abortion rate, % (*n*)	9.2% (7/76)	6.6% (8/121)	*P* = 0.503

*ET, embryo transfer*.

**Table 4:** Reproductive outcomes per FET cycle.

	**Control group** **(***N*** = 100)**	**Surgical group** **(***N*** = 169)**	***P*** **-value**
Number of FET cycles, *n*	102	131	
Clinical pregnancy rate, % (*n*)	38.2% (39/102)	52.7% (69/131)	*P* = 0.028*
Abortion rate, % (*n*)	42.1% (16/39)	26.1% (18/69)	*P* = 0.089
Ectopic pregnancy rate, % (*n*)	0 (0/39)	1.4% (1/69)	*P* = 1.000
Live birth rate, % (*n*)	22.5% (23/102)	38.2% (50/131)	*P* = 0.011*
Premature delivery rate, % (*n*)	0 (0/102)	9.2% (12/131)	*P* = 0.215
Term delivery rate, % (*n*)	21.6% (22/102)	29% (38/131)	*P* = 0.198
Post-term birth rate, % (*n*)	0.98% (1/102)	0 (0/131)	*P* = 0.438
Biochemical abortion rate, % (*n*)	9.8% (10/102)	14.5% (19/131)	*P* = 0.281

*FET, frozen embryo transfer*.

* *P < 0.05*.

Last but not least, the cumulative outcomes after one complete ART treatment for all ET cycles generated from a single stimulation-oocyte retrieval cycle are summarized in Table 5. A total of 178 ET cycles were conducted in the control group and 169 ET cycles in surgical group. The cumulative pregnancy rate (71.0 vs. 59%, *P* = 0.044) and cumulative live birth rate (54.4 vs. 44%, *P* = 0.098) are both higher in the surgical group than the control group, and there was a statistical difference in the cumulative pregnancy rate.

**Table 5:** Cumulative reproductive outcomes from one complete ART cycle including fresh and frozen-thaw ETs.

	**Control group** **(***N*** = 100)**	**Surgical group** **(***N*** = 169)**	***P*** **-value**
Number of ET cycles, *n*	178	252	–
Fresh ET cycles, *n*	76	121	–
FET cycles, *n*	102	131	–
Cumulative pregnancy rate, % (*n*)^a^	59% (59/100)	71.0% (120/169)	*P* = 0.044*
Cumulative live birth rate, % (*n*)^b^	44% (44/100)	54.4% (92/169)	*P* = 0.098

*ET, embryo transfer; FET, frozen embryo transfer*.

a *The percentage of patients who received a clinical pregnancy among all patients entering the stimulus cycle. The numerator is the number of patients with pregnancy obtained after a full cycle, and the upper limit of the per patient's pregnancy number is 1. Only women who either achieved pregnancy or utilized all the embryos resulting from the index stimulation cycle were included in this analysis*.

b *The calculation principle was the same as the cumulative pregnancy rate. Those patients who only obtained clinical pregnancy but did not obtain live birth and still had embryos were not included in the analysis*.

* *P < 0.05*.

According to the AFS classification, we further compared the cumulative pregnancy rate and cumulative live birth rate in the control group (*P* = 0.203 and *P* = 0.427, respectively) and treatment group (*P* = 0.557 and *P* = 0.298, respectively) in patients with complete and partial uterine septum. Either in the control or surgical group, there was no significant difference in the cumulative pregnancy rate and the cumulative live birth rate between incomplete uterine septum and complete uterine septum patients.

## Discussion

The present study was designed to determine whether hysteroscopic metroplasty in women with a septate uterus and secondary infertility improved reproductive outcomes. The results of this study showed that pregnancy outcomes in fresh ET cycles between the two groups have no significant difference. However, the clinical pregnancy rate (52.7 vs. 38.2%, *P* = 0.028) and live birth rate (38.2 vs. 22.5%, *P* = 0.011) of the surgical group were much higher than the control group in FET cycles. Moreover, after analyzing the included data, we found that the rate of patients who had the experience of live birth in the control group was much higher than the surgical group, and statistical differences were observed. This difference means that patients who have had a live birth are more likely to opt out of surgery. We have not found relevant literatures about the comparison of reproductive outcomes between patients who have live birth experience and other secondary infertile patients. However, after adjusting for confounding factors by binary logistic regression, we found that live birth experience had no significant effect on the reproductive outcomes. The proportion of patients who had never had a live birth was higher in the surgical group, but cumulative pregnancy rates were significantly higher in the surgical group than in the non-surgical group. The above results indicate that septum resection in patients with uterine septum and secondary infertility could effectively improve the incidence of good reproductive outcomes.

These results are in agreement with prior studies which showed higher pregnancy rate and delivery rate after treatment. Abuzeid et al. (16) reported significantly higher rates of clinical pregnancy and delivery in the surgical group (including patients who underwent IVF/ET after septal septoplasty or arched hysteroscopic septoplasty) compared to controls with normal endometrial cavities. In addition, the cumulative pregnancy rate and cumulative delivery rate in the surgery group were also significantly higher than those in the control group. Homer et al. (23) also reported an increase in the live birth rate from 3% preoperatively to 80% postoperatively. In another research, 60% of infertile women had pregnancy and 45% had live births after septum incision (24). Furthermore, Mollo et al. (19) conducted a prospective study comparing the pregnancy rates and live birth rates in patients with unexplained infertility with patients with a uterine septum following septum resection. Conception (38.6 vs. 20.4%, *p* < 0.016) and live birth rates (34.1 vs. 18.9%, *p* < 0.05) were significantly higher in women with uterine septum after treatment than in those with a normal uterus. Based on the above research results, the notion that septum adversely affects fecundity and that surgical treatment has a positive effect are supported. However, these findings, including our own studies, have not been confirmed in a prospective RCT. Although many authors recommend further prospective RCTs comparing surgical treatment with no treatment, such studies are difficult to conduct in ethical terms.

Our study has several strengths. Firstly, we calculated the clinical pregnancy rate and delivery rate for both fresh and frozen ET cycles, as well as the cumulative pregnancy rates and cumulative live birth rates after a complete oocyte retrieval cycle, which are more appropriate for evaluating ART outcomes than calculating the indicators for each transplant cycle separately. In addition, owning to recurrent abortion is the main and absolute indication for the treatment of patients with septate uteri (15), when setting the exclusion, we excluded the patients with the history of recurrent miscarriage. In this way, the influence of other unknown factors on reproductive outcome that may lead to recurrent abortion is also avoided. Furthermore, the results of this study not only help to provide better consultation for such patients but can also guide clinicians to take appropriate and effective measures throughout the process.

However, a number of limitations need to be noted regarding the present study. First, the sample size that we included finally was far lower than the theoretical number. The main reasons are that some cases were abandoned because the diagnosis was vague and some patients with secondary infertility and uterine septum gave up transplanting embryo because of Ovarian Hyper-Stimulation Syndrome (OHSS) or dysontogenesis. Second, the hysteroscopic metroplasty of some patients were performed in another hospital, and because of the inconsistency in the technical level, it could interfere with the results. Third, we have attempted to calculate the thickness and depth of uterine septum in order to study its possible influence on the reproductive outcomes. However, only a few hysteroscopic results specifically describe the septum, and the results are not referential. If we have more data in the future, we would like to discuss whether the thickness and depth of the uterine septum affect the reproductive outcomes. One inherent flaw in this study is the retrospective nature of the study, meaning that the findings are less convincing than the results of prospective studies. Therefore, a prospective randomized controlled trial with a large enough sample size is needed to investigate whether surgery improves IVF/ICSI reproductive outcomes in patients with a septate uterus.

## Conclusion

Reproductive outcomes of IVF/ICSI after hysteroscopic correction of uterine septum in women with secondary infertility are better than that of the patients in the untreated group, suggesting that hysteroscopic septum resection can be performed in patients with septate uterus and secondary infertility in order to improve their reproductive performance.

## Data Availability Statement

The original contributions presented in the study are included in the article/supplementary material, further inquiries can be directed to the corresponding author/s.

## Ethics Statement

This study was approved by the Institutional Review Board (IRB) of the Reproductive Hospital affiliated to Shandong University (2019-119). Written informed consent was obtained from the participants when they presented for IVF-ICSI treatment.

## Author Contributions

LY and YC: funding acquisition, conceptualization, and methodology. NZ: supervision and review and editing. SL: data curation. HC and PS: data collection and manuscript writing. All authors contributed to the article and approved the submitted version.

## Funding

This study was funded by the Clinical Research Center of Shandong University (No. 2020SDUCRCC004).

## Conflict of Interest

The authors declare that the research was conducted in the absence of any commercial or financial relationships that could be construed as a potential conflict of interest.

## Publisher's Note

All claims expressed in this article are solely those of the authors and do not necessarily represent those of their affiliated organizations, or those of the publisher, the editors and the reviewers. Any product that may be evaluated in this article, or claim that may be made by its manufacturer, is not guaranteed or endorsed by the publisher.

## Abbreviations

IVF–ET: *in vitro* fertilization–embryo transfer
ICSI: intracytoplasmic sperm injection
ET: embryo transfer
ART: assisted reproductive technology
FET: frozen embryo transfer.
